# The impact of obesity on skeletal muscle strength and structure through adolescence to old age

**DOI:** 10.1007/s10522-015-9626-4

**Published:** 2015-12-14

**Authors:** D. J. Tomlinson, R. M. Erskine, C. I. Morse, K. Winwood, Gladys Onambélé-Pearson

**Affiliations:** Health Exercise and Active Living Research Centre, Department of Exercise & Sport Science, Manchester Metropolitan University, Crewe Green Road, Crewe, CW1 5DU UK; Research Institute for Sport and Exercise Sciences, Liverpool John Moores University, Liverpool, L3 3AF UK

**Keywords:** Ageing, Functional limitations, Obesity, Muscle strength, Sarcopenic obesity

## Abstract

Obesity is associated with functional limitations in muscle performance and increased likelihood of developing a functional disability such as mobility, strength, postural and dynamic balance limitations. The consensus is that obese individuals, regardless of age, have a greater absolute maximum muscle strength compared to non-obese persons, suggesting that increased adiposity acts as a chronic overload stimulus on the antigravity muscles (e.g., quadriceps and calf), thus increasing muscle size and strength. However, when maximum muscular strength is normalised to body mass, obese individuals appear weaker. This relative weakness may be caused by reduced mobility, neural adaptations and changes in muscle morphology. Discrepancies in the literature remain for maximal strength normalised to muscle mass (muscle quality) and can potentially be explained through accounting for the measurement protocol contributing to muscle strength capacity that need to be explored in more depth such as antagonist muscle co-activation, muscle architecture, a criterion valid measurement of muscle size and an accurate measurement of physical activity levels. Current evidence demonstrating the effect of obesity on muscle quality is limited. These factors not being recorded in some of the existing literature suggest a potential underestimation of muscle force either in terms of absolute force production or relative to muscle mass; thus the true effect of obesity upon skeletal muscle size, structure and function, including any interactions with ageing effects, remains to be elucidated.

## Introduction

The prevalence of obesity is a prominent public health concern. Within the UK the proportion of clinically obese adults has increased from 17.5 to 26.1 % between 1996 and 2010 (National Centre for Social Research 2011), and these figures are predicted to rise to 47 % of all men and 36 % of all women by 2025 (Butland et al. 2007). The problem with the rising level of obesity is the associated increased risk in developing a variety of conditions, such as non-insulin dependent diabetes mellitus (DM; Steppan et al. 2001), cardiovascular disease (Larsson et al. 1984), coronary heart disease (Manson et al. 1990), hypertension (Manicardi et al. 1986), stroke (Song et al. 2004) and cancer (Bianchini et al. 2002). In addition to these co-morbidities, obesity has been shown to have a negative impact on skeletal muscle through adolescence (Blimkie et al. 1990; Maffiuletti et al. 2008) to both young (Hulens et al. 2001; Maffiuletti et al. 2007) and old adulthood (Zoico et al. 2004; Rolland et al. 2004).

Researchers have examined the effect obesity has on maximal isotonic (Lafortuna et al. 2005), isometric (Tomlinson et al. 2014a) and isokinetic (Blimkie et al. 1990; Maffiuletti et al. 2007; Hulens et al. 2001, 2002; Delmonico et al. 2009; Hilton et al. 2008) strength in a variety of age classifications ranging from adolescents to the elderly. The majority of these studies with the focus being predominantly in the lower limbs, agree that absolute strength is higher in obese compared to non-obese individuals, and the consensus between all studies is that strength is lower in the loaded musculature when normalised to total body mass. The implications for reduced strength relative to body mass in the lower limbs are foremost relevant to an older population, as these are normally affected by a reduced functional capacity (e.g., difficulty walking, stairs negotiation and rising from a chair or bed) (LaRoche et al. 2011; Rolland et al. 2009; Maden-Wilkinson et al. 2015) and an increased risk of joint pathologies (e.g., knee and hip osteoarthritis) (Cooper et al. 1998; Slemenda et al. 1998) initiated through low muscle strength increasing joint loads (Mikesky et al. 2000) thus increasing the likelihood of developing osteoarthritis and additionally aiding in the progression of the condition, and leading to a reduced quality of life. Accompanying rising age and obesity is the potential increase and effect intramuscular fat has upon muscle mechanics, through lowering muscle quality (Rahemi et al. 2015), however this has not been confirmed in human in vivo research. Therefore, understanding the adaptations of skeletal muscle of individuals who are classified obese across all age groups with specific focus on the elderly needs to be a priority, owing to the combination of a demography of increased prevalence of obesity supplemented with increased life expectancy (Kirkwood 2008).

It is possible that lower relative strength in older obese people when compared to their normal weight counterparts (Tomlinson et al. 2014a) may partly be modulated via a higher state of systemic inflammation (Schrager et al. 2007), as fat deposits can act as endocrine organ secreting various pro-inflammatory cytokines. This hypothesis is supported through obesity-related increases in pro-inflammatory cytokines, specifically interleukin-6 (IL-6) and tumour necrosis factor-alpha (TNF-α; Schrager et al. 2007). Cytokines are in fact associated with lower muscle mass and strength in the elderly (presumably through stimulating muscle protein catabolism and inhibiting muscle protein synthesis) (Visser et al. 2002). These effects may be compounded by impaired skeletal muscle regeneration capacity in obese individuals as hypothesised by Akhmedov and Berdeaux (2013). This has yet to be confirmed in a human population, however animal models have demonstrated an impaired regenerative capacity in obese and diabetic mice (Nguyen et al. 2011) with the mechanism suggested as being through compromised satellite cell function due to lipid overload (Akhmedov and Berdeaux 2013). Yet, the specific effect that chronically high levels of adiposity combined with ageing-associated systemic inflammation and impaired skeletal muscle regenerative capacity may have upon skeletal muscle structure and function is yet to be fully understood.

Therefore, the aim of this review was to examine the known link between adiposity and skeletal muscle force and power generation through adolescence, to young adults and finally old age.

## Does the extra loading of adiposity seen in obesity, act as a training stimulus on skeletal muscle throughout the ages?

**Fig. 1:**
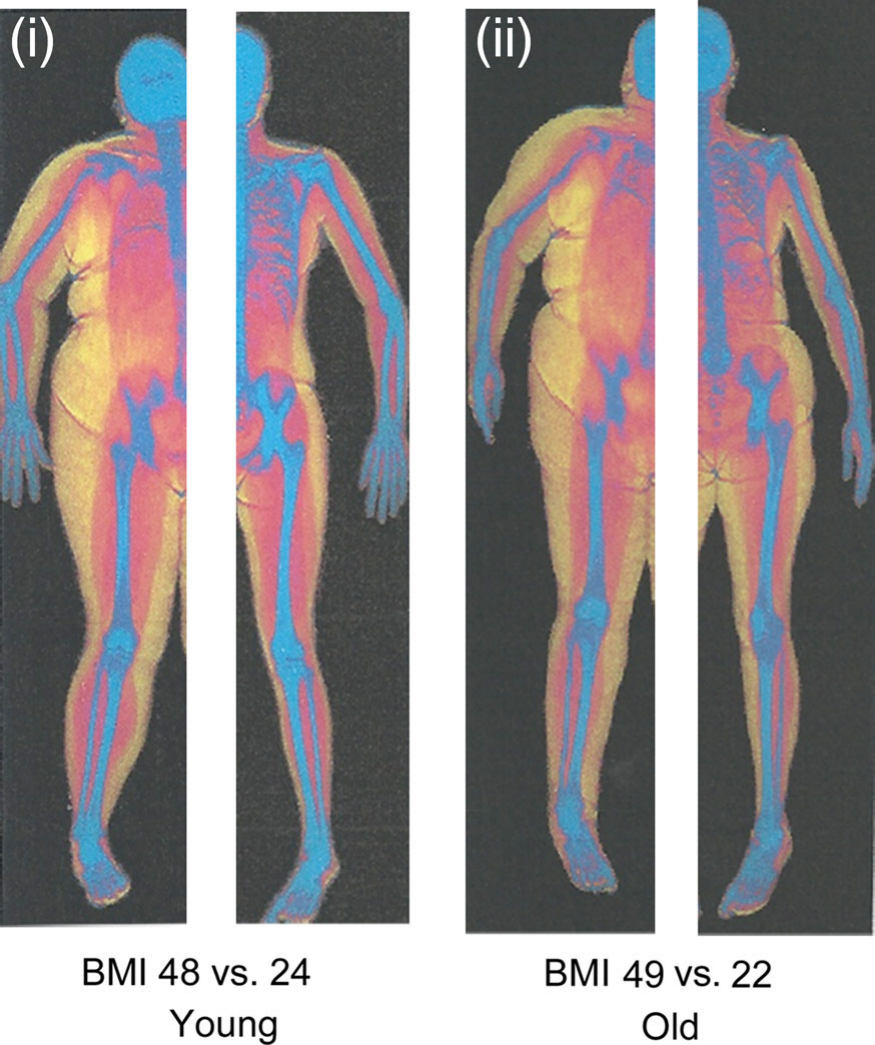
Representative DEXA scans taken from Tomlinson et al. (2014b) of a (**i**) young obese female versus young normal weight female and (**ii**) old obese female versus old normal weight female. *Colour* key: *blue* for bone, *red* for lean tissue, *yellow* for adipose tissue. (Color figure online)

Investigations into the effects of obesity on muscle size and function have described the inter-link between muscle torque and power to body mass, where obese people elicited higher absolute maximum voluntary contraction (MVC) torque and power than normal-weight individuals (Blimkie et al. 1990; Lafortuna et al. 2005; Hulens et al. 2001; Maffiuletti et al. 2007, 2008; Abdelmoula et al. 2012). A rationale for higher absolute MVC torque and power in obese individuals is from the suggestion by Thoren et al. (1973) that extra mass from high levels of fat mass seen in obese individuals might elicit a positive training stimulus on skeletal muscle (see Fig. 1). This hypothesis was strengthened by Bosco et al. (1986) who reported increases in muscle power in anti-gravity muscles following 3 weeks of simulated hypergravity through the use of weighted vests. The weighted vests (7–8 % body mass) utilised in this study were worn from morning until evening similar to the excess (fat) mass an obese individual would carry around daily. It is crucial to note that the duration of this study does not replicate the length of time adiposity acts as a loading stimulus to an obese individual; in addition, all participants were healthy normal weight individuals. This study nonetheless provides a comparable stimulus to the loaded anti-gravity musculature of the lower limbs faced by obese individuals during daily activities for an acute period. The causative explanations given by Bosco et al. (1986) for the increase in performance were through increased motor unit firing rate, additional recruitment of motor units and the synchronisation of these motor units. These specific neural adaptations are accepted to occur in the initial phase of resistance training, with hypertrophy becoming the dominant factor after 3–5 weeks (Moritani and deVries 1979). However, due to the gradual and sustained increases in fat mass that an obese individual would experience, the adaptations to skeletal muscle of an obese individual may differ from that of a healthy individual undertaking loaded resistance exercise. Therefore, this elicits the question that if individuals are obese for numerous years, would this increase muscle strength in the lower limbs through carrying higher inert mass (i.e., adipose tissue) during daily activities?

## The effect of obesity on muscle strength and structure in adolescent individuals

By assessing both neural and muscular components of force generating capacity, Blimkie et al. (1990) were the first to extensively examine skeletal muscle performance in obese and non-obese adolescent males. The main observation from the Blimkie study was lower quadriceps femoris muscle activation in obese compared to non-obese adolescent males (85.1 vs. 95.2 %; 100 % = complete voluntary muscle activation). The obese adolescents studied by Blimkie et al. (1990) were outpatients at a children’s exercise and nutrition centre, while the non-obese adolescents were selected from a local secondary school. It is not known whether the two cohorts were matched for habitual physical activity levels. Any potential differences in physical activity may have explained some of the variability in neuromuscular variables, such as agonist muscle activation and antagonist muscle co-activation (Martinez-Gomez et al. 2011; Moliner-Urdiales et al. 2010; Ramsay et al. 1990), which may have also confounded any potential difference in strength between obese and non-obese boys (Blimkie et al. 1990). Notwithstanding potential differences in the habitual physical activity background of the study participants, the data suggested that relative to their non-obese counterparts, obese adolescents had poorer neural activation capacity, likely leading to a reduction in the degree and/or pattern of muscle fibre recruitment. Yet it has been shown in an adult population that high levels of visceral adiposity is associated with increased neural sympathetic drive (Alvarez et al. 2002).

Interestingly in the Blimkie et al. (1990) study, there were no between group (obese vs. non-obese) differences in absolute isometric strength at a variety of muscle lengths (20°, 40°, 60°, 90° of knee extension) or in isokinetic knee strength (30, 60, 120 and 180°/s). These results differ from later work by Maffiuletti et al. (2008), who reported significantly higher absolute voluntary isometric strength in the obese adolescent at short muscle lengths (+25 % at 40° extension) and during isokinetic efforts (+16 %).

However, the strength in the design of the Maffiuletti et al. (2008) study in comparison to Blimkie et al. (1990) was the control of physical activity in the adolescent males, as the exclusion criteria stated that no individual took part in rigorous physical activity and undertook less than 2 h/week of recreational physical activity. In other words, the fact that Maffiuletti et al. (2008) considered physical activity levels but Blimkie et al. (1990) did not, may in turn account for the disparity in the reported impact of obesity between the two studies. This is due to previously reported data demonstrating that vigorous levels of physical activity can increase strength in the anti-gravity muscles of the lower limb (Moliner-Urdiales et al. 2010). However, Maffiuletti et al. (2008) proposed that a rationale for significantly higher strength at short muscle lengths in the obese adolescent cohort could be their preferentially working at shorter muscle lengths to avoid excessive stress during an activity/sport or to avoid injury. Such a habitual loading protocol would shift the length–tension relationship to the left, hence placing obese adolescents at a disadvantage in daily activities involving a wider range of movement (e.g., deep squatting, getting up from a chair, walking fast, bending).

Similarly to Maffiuletti et al. (2008), others such as Abdelmoula et al. (2012) reported higher absolute maximum isometric knee extension torque (+24 % at 60° of extension) and lower MVC torque relative to body mass (−25 %) in obese compared to non-obese adolescent males. Interestingly, Abdelmoula et al. (2012) reported higher MVC isometric torque normalised to thigh lean mass (+17.9 %) and estimated thigh muscle mass (+22.2 %). This differs from reports by both Blimkie et al. (1990) and Maffiuletti et al. (2008), who reported no significant differences in MVC knee extension torque normalised to quadriceps anatomical cross sectional area (ACSA) (Blimkie et al. 1990) or fat free mass (FFM; Maffiuletti et al. 2008). The discrepancies within these studies may be due to differences in the methodology in assessing thigh/quadriceps muscle mass. Indeed the gold standard in the assessment of muscle size is the physiological cross-sectional area (PCSA) as it accounts for the pennate architecture of the quadricep femoris muscle group. However, Abdelmoula et al. (2012) attributed the higher strength relative to estimated muscle mass in their study sample, to higher agonist muscle activation and lower antagonist muscle co-activation in the obese adolescents. It is noteworthy that whilst Abdelmoula et al. (2012) did measure muscle mass/volume, this could not explain the higher force seen in the obese. It is thus possible that higher muscle activation is indeed a potential explanation but as this was not measured, it remains unclear what explains the higher force in their study. The higher force in the Abdelmoula study may in fact also be due to a greater proportion of faster fibre (Clark et al. 2011). This proposal however is tempered by studies that show there is no real difference in specific tension between slow and fast fibres, and if present this would only explain a small proportion of the difference seen (Ballak et al. 2014). Interestingly, the obese adolescents had lower habitual physical activity levels, which one would normally expect to lead to a lowering in muscle activation capacity (Martinez-Gomez et al. 2011). Indeed as reported earlier, Blimkie et al. (1990) found muscle activation to be significantly lower in obese adolescent boys. Abdelmoula et al. (2012) also proposed that there could have been an increase in the contribution from the synergistic muscles in obese adolescent boys. Whether there may be an obesity-induced alteration in muscle recruitment strategy in young adolescents has yet to be demonstrated. However, it needs to be noted that there is a lack of research examining the neural responses into maximal strength capacity in adolescent obese individuals after controlling for physical activity levels. Further research examining this variable need to investigate both agonist activation using the interpolated twitch technique and antagonist co-activation using surface electromyography to rule out potential differences between obese and normal weight adolescent individuals. In parallel, we would propose an alternative rationale for the higher strength values relative to estimated muscle mass in the Abdelmoula et al. study (2012): the differences in the intrinsic properties of the skeletal muscle of the two cohorts. In support of this hypothesis, previous research demonstrates an increase in fast twitch fibres in obese 26–62 year old adults (Kriketos et al. 1997). The potential shift in fibre type may be explained by the lower physical activity levels of the obese creating an effect similar to what is observed in a detraining model (Staron et al. 1991). Such an effect, however, has yet to be confirmed in an obese adolescent population and in many ways, is opposite to the idea of loading through fat acting as an additional load.

In summary, the general consensus is that obese adolescents exhibit lower relative strength to body mass (see Table 1). Yet discrepancies exist when examining the absolute strength of obese versus non-obese adolescents and strength relative to muscle mass (muscle quality). These differences between studies may be attributed to variability in the methodology, including the control of habitual physical activity difference between participant groups, and/or the methods utilised in the quantification of muscle size. However, current evidence suggests that there is no effect of obesity on muscle quality, but as noted above methodological issues need to be resolved. Interestingly, it has previously been reported that obese adolescents have lower agonist voluntary muscle activation. The implication of this is the potential to underestimate the strength capabilities of obese adolescents in studies not correcting for this variable. Yet, no study to date has examined the effect of antagonist co-activation has upon maximal torque output in obese versus non-obese adolescent individuals thus potentially leading to further underestimating the quality of the muscle exposed to obesity. Further research in adolescents should focus on examining the variables that affect strength production such as agonist muscle activation, antagonist co-activation, PCSA and moment arm length.

**Table 1:** Summarises research conducted into the effect of obesity on muscle strength in adolescence (14–17 years old)

Studies	Gender	Samples (years)	Muscle group	Measures	Findings
Blimkie et al. (1990)	M	11 Obese (16.5) 10 Non-obese (16.6) Range 15–18	KE	– IM KE MVC 90°, 120°, 140°, 160° – IK KE MVC 30°/s, 60°/s, 120°/s, 180°/s – Thigh CSA using CT scans – MUA – Bioelectrical impedance	– IM MVC all angles p = ns – IK MVC all speeds p = ns – IM/BM ↓ obese – IM/CSA all angles p = ns – IK/CSA all angles p = ns – MUA ↓ obese
Maffiuletti et al. (2008)	M	10 Obese (15.6) 10 Non-obese (14.9) Range 13–17	KE	– IM KE MVC 40°, 80° – IK KE MVC 180°/s – Bioelectrical impedance	– IM MVC 40° ↑ obese – IM 40°/FFM p = ns – IM MVC 80° p = ns – IM 80°/FFM p = ns – IK MVC ↑ obese – IK/FFM p = ns
Abdelmoula et al. (2012)	M	12 Obese (14.2) 10 Non-obese (14.4) Range 12–15	KE	– IM KE MVC 60° – DEXA	– IM MVC ↑ obese – IM/BM ↓ obese – IM/FFM p > 0.05 – IM/LM thigh ↑ obese – IM/MM thigh ↑ obese

*M* males, *F* females, *KE* knee extensor, *IM* isometric, *IK* isokinetic, *CSA* cross sectional area, *MUA* motor unit activation, *BM* body mass, *LM* lean mass, *MM* muscle mass, *FFM* fat free mass

## The effect of obesity on muscle strength and structure in young and old adults

One of the first studies to investigate the effects of obesity on muscle strength in an adult population was conducted by Hulens et al. (2001). The authors found that the obese females had significantly higher isokinetic knee extension, trunk extension, flexion and rotational torque than the lean individuals, whilst no impact of obesity was found on handgrip strength suggesting an obesity ‘advantage’ in terms of absolute muscle strength for the loaded musculature. The mean age of the obese cohort was 39 years spanning a large age range (20–65 years), which may have confounded any effect of obesity on skeletal muscle force, as ageing is associated with a decrease in maximal muscle force (Morse et al. 2004). The importance of age classification and its effect on muscle strength is demonstrated in a separate study, in which Hulens et al. (2002) accounted for the confounding age factor and reported that the older obese (41–65 years) had significantly lower knee extension isokinetic MVC torque than their younger obese counterparts (18–40 years).

Hulens et al. (2001) had in fact reported that the loaded antigravity muscles of the knee extensors, back extensors and oblique abdominals were stronger in the obese compared to the lean women. Yet, when normalised to FFM, maximum knee extensor strength was significantly 6–7 % lower in the obese cohort. The discrepancy regarding absolute MVC torque and MVC torque normalised to FFM may be due to lower agonist muscle activation (as seen in adolescents Blimkie et al. 1990). Lower activation of motor units during a maximal contraction would potentially lower maximal strength generation resulting in both lower absolute and normalised MVC. Other explanations for the discrepancy of MVC torque relative to FFM could be the use of FFM instead of muscle volume or PCSA to accurately assess MVC torque relative to muscle size, due to it demonstrating an accurate in vivo representation of the maximum number of parallel-aligned sarcomeres. Interestingly, Hulens et al. (2001) reported no differences in handgrip strength between obese and non-obese individuals opposite to his findings on absolute knee extensor strength, suggesting the additional body mass may act as a training stimulus, i.e., overloading the anti-gravity muscles in a similar way that performance has been shown to increase with the use of a weighted vest (Bosco et al. 1984). Hulens et al. (2001) supported this finding by demonstrating MVC torque relative to FFM during knee flexion was 18–20 % lower in obese versus non-obese individuals, while MVC knee extension torque was only 6–7 % lower, which would suggest that the additional fat mass is a larger training load for the extensors than the flexors. This hypothesis is supported by resistance training being associated with an increase in skeletal muscle specific tension (Erskine et al. 2010).

Lafortuna et al. (2005) went further to examine gender differences in body composition, muscle strength and power output in 95 morbidly obese adults (28 men and 67 women) aged 29 ± 7 years. Body composition was analysed with bioelectrical impedance, while muscle strength of both the upper and lower limbs was assessed using isotonic gym equipment (chest press and leg press) and power output assessed by a standing vertical jump. The main findings of the study by Lafortuna et al. (2005) revealed that obese young men were significantly stronger in both upper and lower limbs and more powerful than the obese young women, and these differences were attributed to greater FFM in the men (77.7 vs. 52 kg), a result which was expected since males tend to demonstrate this effect even in non-obese young adult populations (Janssen et al. 2000). Interestingly, when isotonic strength was normalised to FFM, all differences disappeared between genders in both the obese and normal weight participants. In terms of lower limb power output normalised to FFM, data showed obese males to have lower relative power to FFM than their normal weight counterparts and in addition it showed a strong though non-significant trend for a gender effect (p = 0.059). This could be caused by a change in the intrinsic properties of the skeletal muscle of the lower limb, through a shift in fibre type composition to slower twitch fibres. This further supports the theorem that high body mass loads the antigravity muscles similarly to resistance training, thus causing a fast–slow transition in fibre type composition (Staron et al. 1994). This is demonstrated by a mean difference of 20 kg more inert body mass seen in the obese males than the obese females (128 vs. 108 kg) acting as potential enhanced loading stimulus during daily living activities. The isotonic upper body strength measures reported in this study support this hypothesis, as no differences were reported in upper body strength between normal weight and obese participants irrespective of gender, yet significant strength differences were observed in the anti-gravity muscles during the leg press efforts.

Maffiuletti et al. (2007) reported obese males to have higher absolute torque at all angles and velocities suggesting that, in an adult population, obese individuals do not appear to favourably work at a specific muscle length, contrary to adolescents who exhibit higher absolute strength at short muscle lengths (Maffiuletti et al. 2008). These differences may be explained through obese adolescents preferentially working at shorter muscle lengths as a mechanism to facilitate the accomplishment of habitual daily activities (e.g., shallow/small squats); however this needs further investigation. At their optimal angle and peak velocity obese individuals had 16 and 20 % higher absolute isometric and isokinetic torque, respectively, compared with their non-obese counterparts. Yet, when normalising absolute strength to body mass, maximum isometric and isokinetic knee extension joint torque were respectively 34.5 and 32.5 % lower than in normal-weight individuals similar to previous work in an adult population (Hulens et al. 2001; Lafortuna et al. 2005). However, when both isometric and isokinetic MVC torque were normalised to FFM, any significant differences between cohorts disappeared. It should be noted that the standardisation of MVC torque to total FFM does not differentiate the quadriceps femoris muscle group from other muscle groups, hence extraneous synergistic/antagonistic muscles to knee extension efforts would have confounded the authors’ concluding remarks. Furthermore, MVC joint torque is influenced by additional factors such as voluntary muscle activation capacity, antagonist muscle co-activation and the tendon moment arm (Erskine et al. 2009), which were not considered in this study. To date however, no study has accounted for these differences within the current literature in an adult population when comparing obese–non-obese.

Further research by Maffiuletti et al. (2005) reported that obese individuals have inadequate postural stability when compared to lean persons. These balance issues were improved after a few postural stability-training sessions during a body weight reduction programme. This finding has implications for the prevention of falls, especially in obese elderly individuals who are more at risk of falls and fractures (Himes and Reynolds 2012). Interestingly, the majority of studies investigating the effect of obesity on muscle strength have focussed on the knee extensors. Yet, as demonstrated by Maffiuletti et al. (2005), the contribution of the plantar flexors during postural stability (Onambele et al. 2006) suggests more work should focus on this muscle group when examining the effect of obesity on muscle function.

Hilton et al. (2008) was one of the primary investigators to focus on the plantar flexors. Contrary to the findings of Hulens et al. (2001, 2002), Lafortuna et al. (2005) and Maffiuletti et al. (2007), Hilton et al. (2008) reported that MVC torque and lower limb power were lower in obese compared to non-obese people, both in absolute terms and when power was normalised to muscle volume. Importantly to note, is that the sample size of this study was small (n = 6, BMI = 36 ± 8 vs. n = 6, BMI 28 ± 6) and the obese subjects in this study had DM and peripheral neuropathy. DM is strongly associated with both obesity (Mokdad et al. 2003), and peripheral neuropathy (Young et al. 1993), which is characterised by nerve damage, leading to reduced neural function, motor dysfunction (Andersen et al. 1997) and reduced strength (Andersen et al. 1996). The rationale for lower muscle strength in the obese/peripheral neuropathy cohort of the Hilton et al. (2008) study is likely to be the result of DM-induced neuropathy negatively affecting muscle force generation. Interestingly, individuals only classified obese with no signs of peripheral neuropathy have shown reduced neural function as previously demonstrated by lower muscle activation in both obese versus lean adolescents (Blimkie et al. 1990) and high adiposity versus normal adiposity young adults aged between 18 and 49 years old (Tomlinson et al. 2014a, b, c). Nevertheless more research is necessary to confirm if this is also the case in older adults, and to what degree ageing may impact on any association between obesity and muscle-strength. What is clear however, is that one link is likely to be through increased TNF-α levels in both obesity and insulin resistance (Hotamisligil et al. 1995) which has an apoptotic effect on skeletal muscle tissue (Kewalramani et al. 2010) and would in that manner, negatively impact on the function of the muscle-tendon unit as a whole.

Our own research (Tomlinson et al. 2014a) reported obese adult females (18–49 years old) to have significantly greater plantar flexor strength that their age matched normal and underweight counterparts. This study was the first to control for both antagonist co-contraction and agonist muscle activation during maximal isometric contraction in any age classification. Further research in our group Tomlinson et al. (2014b) also revealed lower maximal strength in an obese adult female cohort when plantar flexion strength was computed relative to gastrocnemius medialis muscle volume. However, after accounting for both the physiological (antagonist co-contraction, agonist muscle activation, PCSA and pennation angle) and biomechanical (moment arm length) determinants of maximal strength capability, all significant differences were removed between both BMI and adiposity classification.

In addition to these findings both Lafortuna et al. (2013) and our own investigations (Tomlinson et al. 2014c) report increasing BMI and adiposity to be associated with increased skeletal muscle volume in a young adult population (18–49 years old), thus explaining a rise in isometric strength demonstrated in the obese (Tomlinson et al. 2014b). Interestingly, Lafortuna et al. (2013) revealed a similar observation as the male obese cohort had greater muscle mass which may be attributed to alterations in the relative levels of cytokines and anabolic hormones (Tipton 2001).

In summary, an analysis of studies in an adult population suggests that obese individuals have significantly higher absolute strength, but lower strength normalised to body mass in the antigravity muscles of the lower limb (see Table 2). However, upper limb strength data reveals no statistical difference between obese and normal weight individuals. This suggests that the loading brought about through higher inert mass (increased adiposity) simulates a resistance-training stimulus, but only specifically to the weight bearing (i.e., antigravity) musculature. Interestingly, when absolute strength measures are made relative to FFM, all significant differences between obese and non-obese cohorts are erased in the majority of cases. However, the use of total body FFM (instead of using PCSA) does not account for the pennate architecture of the knee extensors or plantar flexors, thus potentially confounding the statistical differences between cohorts. Interestingly, the combined effect of obesity coupled with the co-morbidities of DM highlights the detrimental effect of high adipose tissue content in terms of lowering absolute and relative strength through motor dysfunction, and thus negatively impacting on activities of daily living.

**Table 2:** Summarises research conducted into the effect of obesity on muscle strength through young to old adulthood (18–80 years old)

Studies	Gender	Samples (years)	Muscle groups	Measures	Findings
Hulens et al. (2001)	F	173 Obese (39.9) 80 Lean (39.7) Range 20–65	– KE – KF – TE – TF – Forearm	– IK KE MVC 60°/s – IK KF MVC 60°/s – TE 60°/s – TF 60°/s – Handgrip MVC – Bioelectrical impedance	– IK KE MVC 60°/s ↑ obese – IK KF MVC 60°/s p = ns – TE 60°/s ↑ obese – TF 60°/s ↑ obese – Handgrip MVC p = ns
Hulens et al. (2002)	F	241 Obese (39.2) Range 18–65 Two groups Y = 18–40 O = 41–65	– KE – KF – TE – TF	– IK KE MVC 60°/s, 240°/s – IK KF MVC 60°/s, 240°/s – TE 60°/s, 120°/s – TF 60°/s, 120°/s – Bioelectrical impedance – Baeke physical activity questionnaire	– Y obese ↑ IK KE MVC 60°/s, 240°/s – Y obese ↑ IK KF MVC 60°/s, 240°/s – Y obese ↑ TE 60°/s, 120°/s – Y obese ↑ TF 60°/s, 120°/s
Lafortuna et al. (2005)	M and F	28 M Obese (29.2) 8 M NW (30.8) 67 F obese (29.4) 10 F NW (30.0)	– Upper limb – Lower limbs	– CP IT MVC – LP IS MVC	– M ↑ IT CP MVC – M ↑ IT LP MVC – Obese versus NW IT CP MVC p = ns (both M and F) – M obese ↑ IT LP MVC – F obese ↑ IT LP MVC – M obese versus NW IT LP/FFM p = ns – F obese versus NW IT LP/FFM p = ns
Maffiuletti et al. (2007)	M	10 Obese (25.3) 10 Lean (27.0)	– KE	– IM KE MVC 40°, 60°, 80° – IK KE MVC 60°/s, 120°/s, 180°/s – Bioelectrical impedance	– Obese ↑ IM MVC 40°, 60°, 80° – Obese ↑ IK MVC 60°/s, 120°/s, 180°/s – Obese ↑ IM MVC/BM 40°, 60°, 80° – Obese ↑ IK MVC/BM 60°/s, 120°/s, 180°/s – IM MVC/FFM 40°, 60°, 80° p = ns – IK MVC/FFM 60°/s, 120°/s, 180°/s
Hilton et al. (2008)	M and F	6 Obese (58.0: 4 men, 2 women) 6 Overweight (58.0: 4 men, 2 women)	– PF – DF	– PF and DF IM MVC 0° (neutral) – PF and DF IK MVC 60°/s, 120°/s – MV (PF DF muscle group) using MRI – IMAT	– ↓ Obese PF and DF IM MVC 0° – ↓ Obese PF and DF IK MVC 60°/s, 120°/s – Muscle volume p = ns – Obese ↑ IMAT
Lafortuna et al. (2013)	M and F	21 M (50.5, range 31–71) 18 F (55.0, range 32–76)	– Lower limb	– Lower limb MV using CT	– M MV versus adiposity r^2^ = 0.683; p < 0.001 – F MV versus adiposity r^2^ = 0.214; p = 0.05
Tomlinson et al. (2014a)	F	54 Y (26.7) 48 O (65.1) 18 Y Obese (30.9) 11 O Obese (62.5) 13 Y Normal (23.2) 15 O Normal (63.5)	– PF – DF	– PF and DF IM MVC 0° – Co-contraction (using sEMG) – MUA – DEXA	– ↑ Y obese PF and DF IM MVC 0° – O PF and DF IM MVC 0° p = ns – Y co-contraction p = ns – O co-contraction p = ns – Y MUA categorised by BMI p = ns – ↓ Y MUA categorised by body fat % p = ns – O MUA categorised by BMI p = ns – O MUA categorised by body fat % p = ns
Tomlinson et al. (2014b)	F	49 Y (25.5) 45 O (64.8) 16 Y Obese 11 O Obese 12 Y Normal 14 O Normal	– PF	– GM IM PF MVC/MV – GM specific force	– ↓ Y obese GM IM PF MVC/MV – Y GM specific force p = ns – O GM GM IM PF MVC/MV p = ns – O GM specific force p = ns
Tomlinson et al. (2014c)	F	52 Y (25.0) 48 O (65.1) 17 Y Obese (30.9) 11 O Obese (62.5) 13 Y Normal (23.2) 15 O Normal (63.5)	– PF	– GM MV – GM PCSA – GM Lf – GM pennation angle	– ↑ Y obese GM MV – ↑ Y obese GM PCSA – Y GM Lf p = ns – ↑ Y obese GM pennation angle – O GM MV p = ns – O GM PCSA p = ns – O GM Lf p = ns – ↑ O obese GM pennation angle
Zoico et al. (2004)	F	167 F (range 67–78) – NW – Overweight – Obese	– KE	– KE IM MVC	– KE IM MVC p = ns
Rolland et al. (2004)	F	215 Obese (80.0) 630 NW (80.2) 598 Lean (80.7)	– EE – KE	– EE IM MVC – KE IM MVC – Physical activity screen	– Obese ↑ EE IM MVC (both NW and lean) – EE IM MVC p = ns (corrected for PA) – Obese ↑ KE IM MVC 90° versus lean – Sedentary KE IM MVC p = ns – Active obese ↑ KE IM MVC 90°

*M* males, *F* females, *NW* normal weight, *KE* knee extensor, *KF* knee flexion, *IM* isometric, *IK* isokinetic, *IT* isotonic, *CSA* cross sectional area, *MUA* motor unit activation, *BM* body mass, *LM* lean mass, *MM* muscle mass, *FFM* fat free mass, *TE* trunk extension, *TF* trunk flexion, *Y* young, *O* old, *CP* chest press, *LP* leg press, *IMAT* intra muscular adipose tissue, *MRI* magnetic resonance imagery, *CT* computed tomography, *MV* muscle volume, *sEMG* surface electromyography, *GM* gastrocnemius medialis, *PCSA* physiological cross sectional area, *Lf* fascicle length, *PA* physical activity

## The interaction between age and obesity, and its effect on skeletal muscle (sarcopenic obesity)

The age related loss of skeletal muscle mass and function has been termed “sarcopenia” (Narici and Maffulli 2010; Rosenberg 1997). Sarcopenia has been shown to increase the risk of developing functional limitations (e.g., walking and climbing stairs) and physical disabilities as defined by the difficulty in performing daily activities (e.g., shopping, household chores and making meals) (Janssen et al. 2002). Therefore, reversing, delaying, and/or preventing the development of sarcopenia and maintaining functional mobility is paramount to ensuring a good quality of later life. There does not appear to be a single cause for sarcopenia as it is linked with decreased physical activity, chronic systemic inflammation and neuropathic changes leading to motor neuron death and denervation of muscle fibres (Campbell et al. 1973; Degens 2010). However, the presence of obesity coupled with sarcopenia has been shown to exacerbate functional limitations, increasing the difficulty in performing physical functions that require strength (Rolland et al. 2009). Baumgartner et al. (2004) defined the combination of these morbidities as ‘sarcopenic obesity’. Individuals were classified as sarcopenic obese through having an appendicular skeletal muscle mass index [skeletal muscle mass (kg) ÷ stature^2^ (m^2^)] greater than two standard deviations below that of a 20–30 years old young adult reference group (Baumgartner et al. 1998), combined with a body fat percentage above the 60th percentile (Baumgartner et al. 2004).

As discussed above, obesity independent from sarcopenia, has been associated with difficulty in performing daily physical functions such as lifting heavy objects and stair negotiation. Individuals with sarcopenic obesity have an even greater difficulty in performing these daily physical functions (Rolland et al. 2009). Rolland et al. (2009) compared self-reported difficulties with physical functions (i.e., walking, climbing stairs and rising from a chair) in 1308 healthy women aged 75 years old or older. These women were classified into one of four categories (healthy body composition, purely sarcopenic, purely obese and sarcopenic obese). The investigators reported purely sarcopenic women had no increased odds of having physical difficulties with the functional movement assessed when compared to the healthy body composition elderly females of the study. However, the purely obese were reported to have 44–79 % higher probability of having difficulty with the functional movements assessed, whilst the sarcopenic obese had a 2.6 higher probability of difficulty in climbing stairs and 2.35 higher probability of difficulty in going down stairs. Thus, it can be derived from Rolland and colleagues’ study (2009) that sarcopenia and obesity, have synergistic effects when it comes to specific functional movements.

**Fig. 2:**
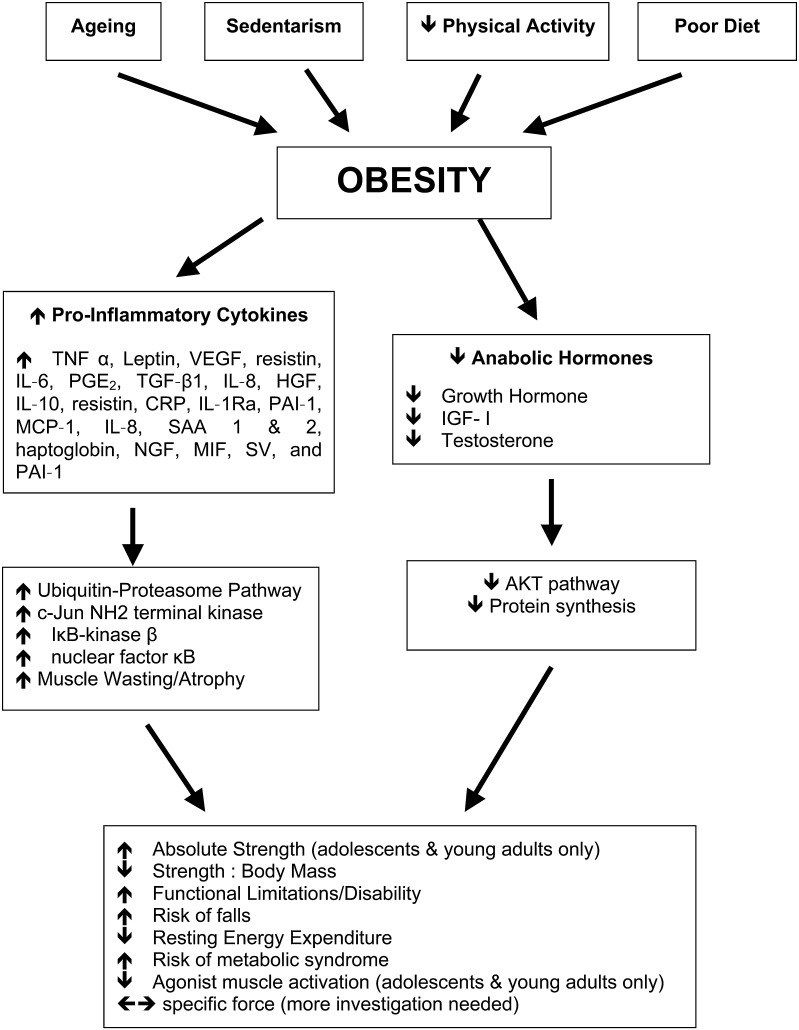
Interplay between obesity, inflammation and skeletal muscle. *Solid arrows* denote events with established evidence. *CRP* C-reactive protein, *HGF* hepatocyte growth factor, *IL-1β* interleukin-1β, *IL-6* interleukin-6, *IL-8* interleukin-8, *IL-10* interleukin-10, *IL-1Ra* interleukin-1 receptor antagonist, *MCP-1*, monocyte chemoattractant protein 1, *MIF* macrophage migration inhibitory factor, *NGF* nerve growth factor, *PGE2* prostaglandin E2, *SAA* *1 and 2* serum amyloid A proteins 1 and 2, *SV* stromovascular, *TGF-β1* transforming growth factor-β1, *TNF-α* tumor necrosis factor-α, *VEGF* vascular endothelial growth factor, *IGF-1* insulin-like growth factor-1

This research was supported by Zoico et al. (2004) who reported older obese women to have a three–four times increased risk of developing functional limitations, where their BMI was higher than 30. However within this study, individuals who had class II sarcopenia (i.e., skeletal muscle mass index 2 standard deviations below a young adult reference group Janssen et al. 2002) had a similar risk of functional limitations as the females who were only characterised as obese. This research suggests that both conditions play a role in limiting physical performance during daily tasks. Potentially also, sarcopenia and obesity may interact, intensifying the unfavourable consequences of the two morbidities. A rationale for the exacerbation of sarcopenia brought about by obesity may be the increased mechanical stress to the musculo-skeletal system through carrying the inert mass of high levels of adipose tissue evident in obesity. In addition, adipose tissue is known to act as an endocrine organ, secreting numerous hormones and inflammatory cytokines (Ahima and Flier 2000), hence enhancing biochemical stress. Obese individuals store chronically high levels of adipose tissue, which causes an increase in circulating pro-inflammatory cytokines (Hotamisligil et al. 1995). Pro-inflammatory cytokines, such as TNF-α (Hotamisligil et al. 1995), IL-1α (Juge-Aubry et al. 2003), IL-6 (Park et al. 2005) and C-reactive protein (CRP) (Park et al. 2005) play a role in cell signalling in the response to both acute and chronic systemic inflammation and can have a detrimental impact on skeletal muscle by stimulating muscle protein degradation (Garcia-Martinez et al. 1993) causing muscle wasting/atrophy and reducing muscle protein synthesis (Mercier et al. 2002). The initiation of muscle wasting/atrophy is modulated via numerous mechanisms such as activation of the ubiquitin–proteasome pathway (Cao et al. 2005; Degens 2010; Saini et al. 2006), which has been shown to be effected via TNF-α (Llovera et al. 1998). Chronically high levels of TNF-α initiates protein degradation and decreased protein synthesis (Mercier et al. 2002), with the net effect being skeletal muscle atrophy (see Fig. 2).

The decrease in protein synthesis can also be related to a reduction in anabolic hormones that would otherwise promote the repair and regeneration of skeletal muscle. This is observed in the reduction in insulin-like growth factor-1 (IGF-1), a promoter of protein synthesis and muscle hypertrophy (DeVol et al. 1990), as reported in severely obese women (Galli et al. 2012). Notably within said study, IGF-1 levels following a surgical intervention (laparoscopic adjustable gastric banding), increased proportionately to the extent of weight loss. This therefore demonstrates that lowering adiposity can improve an individual’s anabolic profile. The inhibition of IGF-1 is thought to be initiated by the TNF-α-mediated activation of Jun N-terminal kinase (JNK) (Grounds et al. 2008). Activation of JNK has also been shown to play a role in the development of insulin resistance and metabolic syndrome through diet induced obesity (Sabio et al. 2010). The overall implications of low IGF-1 levels coupled with elevated pro-inflammatory cytokines in an obese individual would be a blunting of any beneficial effect of enhanced loading. Such an effect may be further exacerbated in an elderly population owing to a less than optimal endocrine milieu normally associated with normal ageing: i.e., low IGF-1, growth hormone and testosterone (Bucci et al. 2013; Lamberts et al. 1997) levels, combined with higher fat infiltration within skeletal muscle (Delmonico et al. 2009) and the ‘inflamed ageing’ phenomenon, i.e., higher circulatory levels of pro-inflammatory cytokines (Visser et al. 2002).

In the literature on the effects of obesity on muscle function in an elderly population, Rolland et al. (2004) examined upper and lower limb muscle strength in obese elderly women and how the effect of habitual physical activity levels contributed to any differences in maximum muscle strength between active/non-active obese elderly individuals. The study consisted of three cohorts: (i) obese (n = 215, BMI = 31.9), (ii) normal weight (n = 630, BMI = 26.3), (iii) lean (n = 598, BMI = 21.6) participants (it should be noted that participants ought here to have been categorised as obese, overweight and normal weight individuals, to be more correct on the terminology). Physical activity was controlled for and defined as being active by taking part in at least one recreational physical activity (i.e., hiking, swimming and gardening) for greater than 1 h/week. The obese individuals were shown to be less physically active than both the lean and normal weight cohorts, yet when classifying participants as either sedentary or active, obese individuals with high activity levels demonstrated higher absolute isometric knee extension strength when compared to lean individuals. However, when individuals were classed as sedentary, any significant differences between cohorts were eradicated in relation to knee extension strength. Interestingly, there was no difference in handgrip or elbow extension strength between cohorts even though obese individuals had significantly larger arm muscle mass when compared to categorised normal weight and lean individuals (Rolland et al. 2004). This may be explained by the hypothesis presented earlier regarding elevated adiposity causing additional overloading of the anti-gravity muscles (e.g., quadriceps, triceps surae) during routine daily activities, e.g., walking, climbing steps, etc. This creates an environment of hypergravity, which has been shown to increase isokinetic plantar flexor strength by 40 % in post-menopausal women following 12 weeks of resistance training using weighted vests (Klentrou et al. 2007).

This benefit of weighted exercise has also been shown in women aged between 50 and 75 years, who saw increases in muscle strength, power and lean leg mass after a 9-month training regime (Shaw and Snow 1998). In addition to these variables, the participants’ postural stability was improved in the medio-lateral direction. This specific improvement in postural balance has been shown to benefit elderly frail individuals, as most falls occur in a medio-lateral plane (Greenspan et al. 1998). Contrary to this beneficial effect of weighted exercise, obese individuals are shown to have poor postural stability (Maffiuletti et al. 2005). Thus, whilst comparisons may be made between the additional loading of a hypergravity environment against the excess loading experienced by an obese individual (through their own body mass), the detrimental consequences of obesity appear to outweigh any potential benefits of increased loading. However, as shown by Rolland and colleagues (2004), increasing physical activity levels may potentiate an increase in muscle strength thereby lessening the detrimental consequences of obesity. Interestingly in support of this idea, our own work (Tomlinson et al. 2014a) reveals that after controlling for physical activity levels, no differences in raw plantar flexor muscle strength exist between obese versus normal elderly females aged between 50 and 80 years old, when strength is corrected for antagonist co-contraction and agonist muscle activation.

Villareal et al. (2004) looked into the association between physical frailty and body composition in obese elderly (n = 52), non-obese frail elderly (n = 52) and non-obese non-frail elderly (n = 52). The classification of physical frailty was defined using three specific tests: a modified physical performance test (PPT) consisting of 7 standardised timed tasks (such as a 50 feet walk, putting on and removing a coat, standing up from a 16 inch chair 5 times and climbing a flight of stairs), Peak aerobic power (VO_2_ peak) using a graded treadmill test and a log of their activities during daily living. From these three tests, physical frailty was then defined if participants met two out of three of the following criteria: modified PPT score of between 18 and 32, VO_2_ peak of 11–18 mL/Kg/min and difficulty in performing two daily activities (Villareal et al. 2004). Within the study it was reported that the obese elderly individuals had greater absolute FFM than both non-obese frail and non-obese non-frail cohorts, yet when normalised to total body mass it was found to be lower. In addition, the obese individuals had poorer muscle quality, i.e., lower knee extension strength relative to leg lean mass, compared to their non-obese counterparts. A limitation of this assertion is that dual energy x-ray absorptiometry (DEXA), as opposed to magnetic resonance imagery (MRI) or ultrasound, cannot differentiate between muscle groups. This is important, due to the potential error in relating the torque produced to whole leg lean mass instead of the muscle group undertaking the specific task.

Delmonico et al. (2009) examined the effects of sarcopenic obesity on muscle strength and physical function. They reported an age-related increase in intramuscular fat content at mid-thigh in both men and women. Due to this being a longitudinal study it was reported that after 5 years, intramuscular fat content increased irrespective of changes in body mass and subcutaneous fat in the thigh. Coupled with the increase in intramuscular fat, the data showed that the loss of knee extensor strength was two–five times greater than the loss of mid-femur muscle cross sectional area with ageing (Delmonico et al. 2009). The higher reported decrease in strength may have partly been explained by lower muscle activation and/or an increase in co-activation, which was not accounted for when measuring maximal strength. This study demonstrates that the loading effect seen in younger individuals does not attenuate the age-related loss of strength. The disproportionate loss of strength versus muscle mass was suggested through a loss in muscle quality, which has previously been reported by both Morse et al. (2005) and Goodpaster et al. (2006) in an elderly cohort. These studies suggest that the rate of force loss with ageing is similar in both obese and non-obese persons. It would not be unreasonable to expect the positive association between recreational physical activity and lower limb strength in elderly obese individuals (Rolland et al. 2004) to offset a decrease in muscle quality.

In summary, with the increase in life expectancy and the rise in obesity, it is unsurprising that sarcopenic obesity incidence is also increasing (James 2008). Whilst the body of information regarding the effects of sarcopenic obesity on skeletal muscle structure and function is increasing, there remain gaps in our knowledge. In contrast, ageing has been associated with lower agonist muscle activation (Morse et al. 2004), an increase in antagonist muscle co-activation (Klein et al. 2001), a decrease in muscle fascicle pennation angle (Morse et al. 2005) and lower muscle volume (Thom et al. 2005). To-date, these adaptations have not been systematically examined in a sarcopenic obese elderly population. However, community dwelling obese adult females have shown similar characteristics when compared to a young adult obese female population (Tomlinson et al. 2014a, b, c), obesity was shown to exacerbate the age-related physical function limitations associated with the loss of muscle mass and strength.

## Conclusion

Obesity is recognised as being a worldwide epidemic and a major public health concern (James 2008). It has been reported to have detrimental implications for the functioning of skeletal muscle yet very little is known about the specific adaptations of skeletal muscle by gender and age, in the presence of chronically elevated adiposity.

The consensus within the literature is that obese individuals have reduced maximum muscle strength relative to body mass in their anti-gravity muscles compared to non-obese persons (Abdelmoula et al. 2012; Blimkie et al. 1990; Hulens et al. 2001; Lafortuna et al. 2005; Maffiuletti et al. 2007, 2008; Rolland et al. 2004; Delmonico et al. 2009). This effect on an obese individual is shown to increase the risk of developing osteoarthritis (Slemenda et al. 1998) and potentially cause functional limitations especially in the elderly (Visser et al. 2005). Evidence suggests that high levels of adiposity may impair agonist muscle activation in the young (Tomlinson et al. 2014a), adding to or perhaps leading to the functional limitation of low strength relative to body mass.

Future research is needed to systematically investigate whether body fat percentage per se may be related to agonist muscle activation (using the interpolated twitch technique) and antagonist co-activation (using surface electromyography) and/or morphological characteristics, such as muscle volume, PCSA and architecture (using gold standard techniques such MRI, computed tomography and ultrasound imaging) in the elderly focusing on individuals who are classified sarcopenic obese. Interestingly within this age classification, there appears to be a lack of longitudinal studies examining how physical/sedentary activity across the age span impacts upon the aforementioned variables. When examining the design of future work, classification of obesity should be made by adiposity (using DEXA) instead of classification by individuals BMI and classification of sarcopenia by the appendicular skeletal muscle mass index. Key also, is to determine the impact of duration of obesity, on the reported musculo-skeletal structural and functional characteristics presented during the study. Such knowledge would aid in the development of therapeutic targets.
